# Study on mechanical properties and microstructure of soil-cement solidified recycled aggregate concrete using bridge demolition waste

**DOI:** 10.1371/journal.pone.0343109

**Published:** 2026-03-16

**Authors:** Yang Du, Ziqing Cheng, Peichen Cai, Ke Lou, Shaodong Wei, Xiaoyu Wang, Shengjie Wang, Xuesong Mao, Feiheng Huang, Qian Wu

**Affiliations:** 1 Shandong Expressway Construction Management Group Co., Ltd., Jinan, China; 2 Shandong Expressway Yanpeng Highway Co., Ltd., Yantai, China; 3 College of Highway, Chang’an University, Xi’an, China; Shandong University of Technology, CHINA

## Abstract

This study investigates the mechanical behavior and microstructural characteristics of recycled aggregate concrete (RAC) solidified with soil cement, using high-quality recycled aggregates derived from demolished reinforced-concrete bridges. Concrete mixtures were prepared with varying recycled aggregate replacement ratios (60%, 80%, 100%) and soil-cement contents (15%, 18%, 21%). Compressive strength (CS) tests and scanning electron microscopy (SEM) analyses were conducted. Owing to the dense and high-strength nature of bridge-demolition aggregates, increasing the recycled aggregate replacement ratio from 60% to 100% led to a noticeable improvement in compressive strength—an outcome that contrasts with most studies using lower-grade construction waste. The results showed that increasing the soil-cement content raised the 28-day CS to a maximum of 21 MPa. The 7-day CS exceeding 70% of the 28-day value, indicating rapid early strength development. Compared with conventional concrete, the RAC incorporating bridge-derived aggregates demonstrated enhanced crack resistance and a denser microstructure. A regression model relating mix parameters and curing age to CS achieved coefficients of determination exceeding *R*^2^ = 0.99, demonstrating its strong predictive capability. SEM observations showed progressive pore refinement and matrix densification during curing, consistent with the measured strength gains. These findings support the engineering application of RAC and promote the sustainable reuse of construction waste in infrastructure projects.

## Introduction

With the rapid advancement of highway reconstruction and expansion projects, a large quantity of construction waste—including discarded concrete from demolished bridges and buildings—is being generated [1,2]. To enhance the recycling and reuse of this construction waste, it is feasible to process it into recycled concrete or other building materials for engineering applications [3,4]. However, due to the diverse sources of construction waste, the resulting recycled concrete exhibits considerable variability in mechanical strength. For instance, construction waste from brick-concrete structures tends to have low strength and can only partially replace natural aggregates. In some cases, additional reinforcement treatment is required, which significantly increases production costs [5]. In contrast, demolition waste from bridge structures typically originates from high-strength concrete. The crushed aggregates from these materials can be entirely reused in recycled concrete production, thereby substantially reducing costs [6]. Furthermore, recycled concrete production generally involves binders to consolidate the construction waste. Unlike traditional cement or lime binders, soil cement is a new type of green material made from industrial waste such as red mud, fly ash, and coal gangue. The utilization rate of industrial waste residue in its production process exceeds 80% [7]. As a result, soil cement presents a promising alternative to conventional binders for recycled aggregate preparation. In addition to the above, recent international research has increasingly emphasized the sustainability, carbon reduction potential, and resource efficiency of incorporating multi-source waste materials into concrete. Numerous studies have demonstrated that waste glass powder, recycled concrete fines, waste plastics, and supplementary cementitious materials can reduce clinker consumption while maintaining structural performance [8,9]. Globally, the recycling of construction and demolition waste (CDW) has become a key strategy for achieving circular economy goals, with the European Union, the United States, and China all reporting significant research progress on structural applications, durability enhancement techniques, and environmental life-cycle assessment of recycled concrete [10,11]. These studies collectively highlight that the technical feasibility of CDW reuse must be evaluated alongside environmental benefits and cost-effectiveness, forming a more holistic performance framework.

In recent years, many researchers have investigated the reuse of construction waste. Through mechanical tests, they have validated the basic physical performance of these materials and compared the curing effects of various binders under different experimental conditions. In terms of the mechanical performance of recycled aggregate concrete (RAC), Xiao et al. [12] studied the influence of different aggregate systems and found that although the strength development trend of RAC was similar to that of ordinary concrete, RAC exhibited reduced peak stress and increased peak strain. González et al. [13] evaluated the mechanical performance of RAC and reclaimed asphalt pavement (RAP), concluding that when 3/4-inch recycled aggregates were used, both RAC and RAP could serve as viable alternatives for porous pavements in pedestrian and light traffic zones. Zhao et al. [14] developed a brick aggregate-reed straw concrete and proposed a conversion formula between compressive and tensile strength. Microscopic observations revealed that the interfacial transition zone was a weak point in RAC. Chen Shoukai et al. [15] tested RAC specimens with five replacement rates and found that peak stress initially increased and then decreased with higher replacement rates, while the elastic modulus declined and the peak strain rose. Liu et al. [16] also confirmed that increasing the proportion of recycled coarse aggregates led to a reduction in the mechanical performance of RAC. However, while the above works provide valuable mechanical insights, a critical review reveals several limitations. Most studies focus on compressive or tensile behavior but seldom address long-term durability, field constructability, cost–benefit considerations, or barriers to engineering implementation. International studies further indicate that variability in CDW sources, insufficient quality control of recycled aggregates, and uncertainty regarding binder–aggregate interactions remain major challenges limiting full-scale practical adoption [17, 18]. Therefore, research must not only document mechanical performance but also clarify the mechanisms that control performance variability and identify pathways to improve reliability for real engineering applications. Together with the aforementioned studies on multi-waste powders and waste glass–based systems [8,9], these works highlight that the reuse of different solid wastes in concrete is not only a structural engineering problem but also a key pathway for environmental impact reduction.

In terms of soil-based or waste-based cementitious stabilization of soils and recycled materials, Xie et al. [19] conducted an experimental study using a fully industrial solid waste–based cementitious binder to stabilize aeolian sand, demonstrating that the waste-derived binder could achieve CS and microstructural densification comparable to conventional cement-stabilized sand. Similarly, Bai et al. [20] used a fly ash + GGBFS–based geopolymer binder to treat aeolian sand for subbase applications, obtaining satisfactory strength, CBR, and lower carbon footprint compared to cement-stabilized variants. More broadly, Leon [21] explored the use of quarry dust and recycled concrete aggregate fines to stabilize expansive clay subgrade, showing improved compaction, strength, and reduced swelling — indicating that recycled waste materials combined with modest binder doses can effectively function as soil stabilizers. These studies, by replacing or reducing traditional cement with industrial by-products or recycled aggregates, support the feasibility and environmental advantages of soil-stabilization approaches based on waste materials, and provide a foundation for extending such methods to high-strength recycled aggregates derived from demolished concrete structures. Nevertheless, existing studies on soil cement primarily concentrate on soil stabilization, with very limited international research exploring its interaction mechanisms with high-strength recycled aggregates. This represents a critical gap because recycled aggregates derived from demolished bridge structures—unlike soil or low-strength CDW—exhibit distinct microstructural characteristics, strong angularity, and high intrinsic strength. These attributes may fundamentally alter hydration, bonding, and densification processes when soil cement is used as the binder. Understanding these mechanisms is essential for reliable practical implementation in subbases, backfills, and sustainable pavement materials.

Thus, this paper designs two groups of comparative tests to analyze the mechanical strength formation mechanism of soil cement solidified recycled aggregate. The first group uses soil cement with different dosages of the same recycled aggregate to solidify, and analyzes the strength change law of recycled aggregate concrete with different dos-ages of soil cement over time. The second group uses soil cement with the same dosage of different recycled aggregate dosages to solidify, and analyzes the strength change law of recycled aggregate concrete with different dosages of recycled aggregate over time. On this basis, microscopic scanning electron microscopy (SEM) tests are performed to analyze pore evolution, hydration product density, and microstructural changes.

By integrating mechanical response with microstructural evidence under a waste-based binder system, this study aims to (i) clarify the fundamental mechanisms governing strength variability, (ii) provide guidance for optimizing soil-cement mix designs for high-quality recycled aggregates, and (iii) address practical engineering concerns such as sustainability benefits, material cost reduction, and applicability in large-scale highway reconstruction. These aspects directly respond to the identified research gap and improve the feasibility of applying soil-cement–stabilized recycled aggregates in real-world infrastructure projects.

## Materials and methods

### Test materials

**Fig 1:**
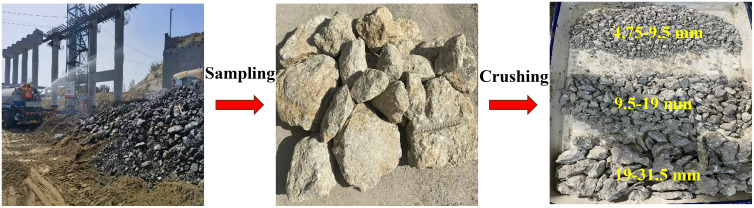
Process of obtaining recycled aggregate raw materials.

The raw materials used in the concrete (design strength C15) prepared in this study include soil cement, natural coarse/fine aggregate, recycled aggregate, water reducer, and so on. Among them, the soil-cement binder used in this study is a soil tuff, an eco-friendly cementitious material produced from industrial by-products such as steel slag, coal gangue, fly ash, and red mud. Its basic physical properties are as follows: density 2850 kg/m^3^, specific surface area 600 cm^2^/g, and compressive strengths of 12.5 MPa (3 days) and 48.5 MPa (28 days). XRD analysis shows that soil cement is primarily composed of SiO_2_, CaO·Al_2_O_3_·2SiO_2_·4H_2_O, and Al_2_O_3_, with additional Fe_2_O_3_ originating from industrial waste residues. These results provide a clear chemical and physical characterization of the soil-tuff binder. Compared with the use of traditional cement, it can greatly reduce the carbon footprint of buildings and achieve additional economic benefits [22]; the natural fine aggregate is river sand with a fineness modulus of 2.70 and an apparent density of 2590 kg/m^3^; the natural coarse aggregate is natural crushed stone sand with a particle size of 4.75–31.5 mm; the recycled aggregate is selected from waste concrete blocks with a design strength of C40, which are mainly from the demolition of the Shandong Yanpeng Expressway Bridge. The surface of the concrete block is rough, with obviously hardened cement mortar and small stone chips attached to its surface. After being crushed by a crusher, screened, cleaned, and dried, the recycled aggregate with a continuous gradation of 4.75–31.5 mm is obtained, as shown in Fig 1; the water reducer is polycarboxylic acid high-performance water reducer HPWR-S, with a water reduction rate of up to 25%; the water is laboratory tap water. In addition, to clarify the difference in mechanical properties between concrete from abandoned bridge building demolition and traditional graded crushed stone, the selected recycled aggregate and graded crushed stone were subjected to water absorption, crushing value, and point load tests. The specific test results are shown in Table 1. As can be seen from Table 1, the strength of the recycled aggregate used in this study is slightly greater than that of the traditional graded crushed stone, which is different from other studies [23]. This is because the recycled aggregate used in this paper comes from the crushed material of high-strength concrete (all above C60) such as abandoned bridge building demolition. Therefore, even if it is the crushed material after demolition, the test results show that its strength is still relatively high.

**Table 1:** Comparison of physical and mechanical properties between recycled aggregate and graded crushed stone.

Performance Indicators	Recycled aggregates	Graded gravel
**Bulk density/(kg·m**^**-3**^)	1725	1641
**Apparent density/(kg·m**^**-3**^)	2859	2704
**Crushing value/%**	16.46	17.15
**Point load strength/(MPa)**	6.34	5.10
**Water absorption/%**	5.70	6.52

Note: All data presented were obtained from the authors’ laboratory tests.

### Mix design of recycled aggregate concrete

To investigate the effects of soil cement content and recycled aggregate replacement ratio on the mechanical properties of recycled aggregate concrete (RAC) stabilized with soil cement, this study refers to the *Ordinary Concrete Mix Design Code* (JGJ 55–2011) and the *Recycled Concrete Mix Design Code* (DB37-T5176-2021) to prepare concrete with a target strength grade of C15. In each mix, the coarse aggregate is composed of recycled aggregate and natural graded crushed stone. The soil cement content is set to 15%, 18%, and 21%, which refers to the mass percentage of soil cement relative to the total mixture (by mass). The remaining 85%, 82%, and 79%, respectively, consists of coarse aggregates, including recycled aggregate (RA) and natural graded crushed stone (GCS). Within this coarse aggregate portion, the recycled aggregate replacement ratio is defined as the mass fraction of RA relative to the total coarse aggregate. For example, in the T15-R60 mix, the soil cement accounts for 15% of the total mass, and the coarse aggregate accounts for 85%. Within this 85%, recycled aggregate constitutes 60%, and natural gravel constitutes 40%. In addition, to ensure consistent gradation across mixes, both recycled and natural aggregates are proportioned to maintain the same particle size distribution: 4.75–9.5 mm, 9.5–19 mm, and 19–31.5 mm in a fixed mass ratio of 1:1.5:1.5. Table 2 lists the specific mix design details for each cubic meter of recycled aggregate concrete.

**Table 2:** Mix design of recycled aggregate concrete (kg/m^3^).

No.	Soil cement	Sand	Recycled aggregate	Graded gravel	Water	Water reducing agent
**T15-R60**	359	822	583	388	204	3
**T15-R80**	359	822	777	187	204	3
**T15-R100**	359	822	971	0	204	3
**T18-R100**	431	771	937	0	244	3
**T21-R100**	503	720	903	0	285	3

### Experimental methods

**Fig 2:**
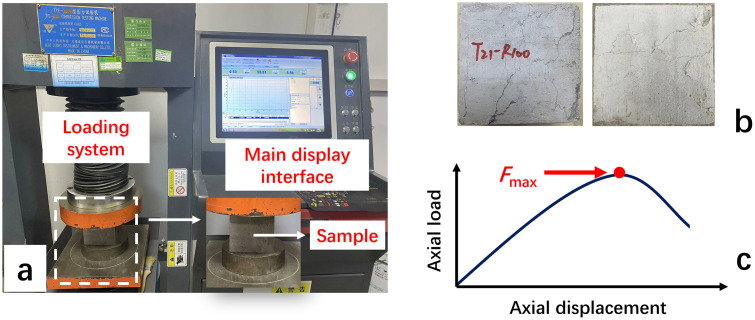
Compressive strength test process [24]. (a) Universal press machine; (b) Compressed test specimen; (c) Typical force-displacement curve.

In this study, the compressive strength test of the prepared recycled aggregate concrete (100 mm × 100 mm × 100 mm) was carried out according to the Standard for Test Methods of Mechanical Properties of Ordinary Concrete (GB/T 50081−2019) (Fig 2(a)). The compressive strength loading rate was set to 0.5kN/s, and the results were accurate to 0.1MPa. The compressive strength of the test blocks under different mix ratios (Table 3) at standard curing ages of 7d, 14d, and 28d were tested respectively. The compressive strength of the recycled concrete under different soil cement content and different recycled aggregate replacement rates was obtained by analyzing the stress-strain curve during the compression process of the test blocks (Fig 2(b)), and the strength difference of concrete with different recycled aggregates was compared and analyzed. Subsequently, the apparent crack analysis was carried out on the loaded test blocks, and the failure characteristics of the concrete were analyzed according to the development of the cracks (Fig 2(c)). Finally, the internal structure of the test blocks after loading and destruction was taken for SEM microstructure analysis to deeply understand the density of the mutual cross-linking of the internal structure of the concrete, to explain and predict its macroscopic mechanical behavior.

**Table 3:** Quantitative crack morphology parameters of recycled aggregate concrete at different curing ages.

Category	Number of cracks	Total crack area	Average crack width	Crack density	Crack area ratio
7d	50	337.55 mm^2^	2.28 mm	0.0320 mm^-1^	0.0338
14d	61	299.04 mm^2^	1.88 mm	0.0328 mm^-1^	0.0299
28d	163	237.88 mm^2^	0.82 mm	0.0678 mm^-1^	0.0238

## Physical and mechanical properties of soil cement solidified recycled aggregate concrete

### Analysis of apparent crack failure morphology

**Fig 3:**
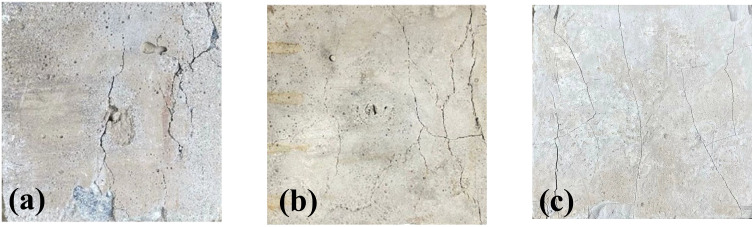
Distribution of apparent cracks in recycled aggregate concrete under compression at different curing ages. (a) Curing age 7 days; (b) Curing age 14 days; (c) Curing age 28 days.

**Fig 4:**
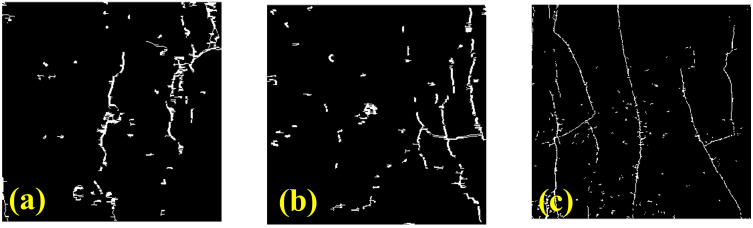
Schematic diagram of apparent crack extraction of recycled aggregate concrete under compression at different curing ages. (a) Curing age 7d; (b) Curing age 14d; (c) Curing age 28d.

In the compressive strength test of recycled aggregate concrete cured with soil cement, the specimens at different curing ages showed different crack morphologies. Fig 3 shows the distribution of apparent failure crack morphology of recycled aggregate concrete at 7 days, 14 days, and 28 days. Here, the soil cement content is 15% and the replacement rate of recycled aggregate is 100%. On the basis of the images in Fig 3, MATLAB image-processing scripts were used to segment the surface cracks and to extract quantitative crack-morphology parameters, including the number of cracks, the total crack area, the average crack width, the crack density (defined as the total crack length per unit projected area), and the crack area ratio. The extracted crack maps are shown in Fig 4, and the corresponding numerical values for the three curing ages are summarized in Table 3. As shown in Fig 3(a) and Fig 4(a), the crack morphology of the 7-day recycled concrete sample is characterized by a relatively obvious longitudinal main crack, with a large crack width and a deep extension. Consistent with this observation, Table 4 shows that the 7-day specimen has 50 cracks, the largest total crack area (337.55 mm^2^), the highest average crack width (2.28 mm) and the largest crack area ratio (0.0338). This phenomenon indicates that the cohesion of the sample is low at this time, and the bonding degree inside the concrete has not yet reached the optimal state, resulting in cracks that are easy to generate and expand along the weaker interface during compression. In addition, there may be large particles of recycled aggregate at the starting point of some cracks. These large particles of recycled aggregate have poor interface bonding with the surrounding matrix, becoming stress concentration points, which in turn lead to the formation of cracks. Fig 3(b) and Fig 4(b) shows a 14-day recycled concrete sample. Compared with the 7-day sample, the number of cracks in the 14-day sample is slightly increased, but the crack width is relatively small and the distribution is more uniform. Quantitatively, the 14-day specimen exhibits 61 cracks and a similar total crack area (299.04 mm^2^), while the average crack width decreases to 1.88 mm; at the same time, the crack density increases to 0.0328 mm^−1^, indicating a denser network of relatively narrow cracks (Table 3). This shows that with the extension of curing time, the cohesion of the recycled concrete is improved, the integrity of the material is improved, and the compressive strength is gradually enhanced. However, due to the strength of the recycled aggregate itself and the defects in the interface area, the cracks are still concentrated in these relatively weak areas. At this stage, the crack expansion mode develops from a single main crack to a network structure of multiple fine cracks, reflecting the change in the internal stress distribution of the recycled concrete. Besides, Fig 3 (c) and Fig 4(c) shows a 28-day recycled aggregate concrete sample and the cracks at 28 days are more subtle and evenly distributed. As listed in Table 3, the 28-day specimen has 163 cracks but the smallest total crack area (237.88 mm^2^) and crack area ratio (0.0238), together with the lowest average crack width (0.82 mm). This crack morphology shows that the mechanical properties of recycled concrete approach their stable state after 28 days, and the density and uniformity of the internal structure are significantly im-proved. Although there are many cracks, the influence of cracks on the overall compressive properties of the material is reduced because the cracks are small and interpenetrating. The crack characteristics at this stage also reflect the strengthening effect of the interface transition zone between the recycled aggregate and the matrix, indicating that the interface area has been improved to a certain extent.

**Table 4:** Fitting surface parameter.

Parameter	Eq. (1) fitting parameter value	*R* ^2^	Eq. (2) fitting parameter value	*R* ^2^
α	0.01667	0.9984	6.67 × 10^−4^	0.9953
β	−4.308 × 10^−3^		−0.6122 × 10^−3^	
γ	1.599 × 10^−2^		3.163 × 10^−3^	
δ	−0.3667		−0.06667	
λ	0.1551		0.1088	
σ0	11.44		10.42	

### Stress-strain curve analysis

**Fig 5:**
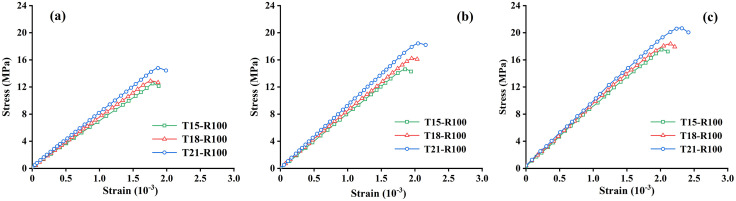
Stress-strain curves of recycled aggregate concrete with different soil cement content. (a) 7d curing age; (b) 14d curing age; (c) 28d curing age.

**Fig 6:**
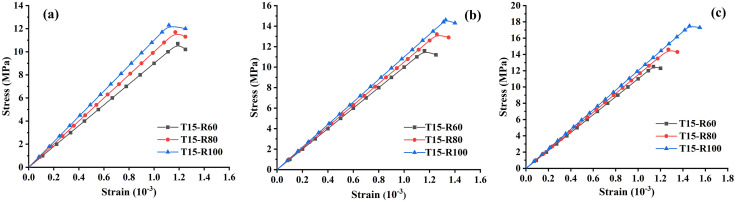
Stress-strain curves of recycled concrete under different recycled aggregate replacement rates. (a) 7d curing age; (b) 14d curing age; (c) 28d curing age.

Figs 5 and 6 show the stress-strain curves of recycled aggregate concrete at different curing ages under different soil cement content and recycled aggregate replacement rates. The stress-strain curve in Fig 5 reveals that with the increase of soil cement content, the compressive strength of recycled aggregate concrete shows significant differences at different curing ages. When the content of cement content exceeds 18%, the recycled aggregate concrete shows a higher peak stress, and the recycled aggregate concrete reaches 16 MPa at the curing age of 14 days, meeting the strength requirements of the design requirement C15, indicating that soil cement can effectively improve the mechanical properties of concrete within this content range. In addition, from the curing age of 7 days, 14 days to 28 days, the peak value of the stress-strain curve of concrete gradually increases, especially at 28 days. This shows that with the extension of curing time, the hydration reaction inside the concrete is more complete, the structure is more compact, and the compressive strength of the material is significantly improved. Based on the analysis of Fig 5, the mechanical properties of recycled aggregate concrete can be optimized by properly controlling the amount of soil cement (such as controlling it between 18% and 21%) and fully considering the curing age, thereby improving its reliability and durability in engineering applications. Fig 6 shows the effect of different recycled aggregate replacement rates on the stress-strain curve of recycled aggregate concrete. As the recycled aggregate replacement rate increases, the peak strength of the stress-strain curve increases, especially at high replacement rates (such as 100% replacement). This trend is particularly obvious. The reason for this is that the recycled aggregate used in this study comes from the high-strength concrete crushed material of abandoned bridges. The test results show that its physical and mechanical properties are stronger than those of graded crushed stone (Table 1), which leads to the enhancement of the overall mechanical properties of the replaced concrete. That is, the recycled aggregate used this time comes from the abandoned bridge demolished by the Shandong Expressway, and its original strength is significantly higher than that of ordinary crushed stone [23]. Even after crushing, these recycled aggregates still maintain a high strength level. It is this characteristic that leads to the above phenomenon. Compared with recycled aggregates mixed with crushed stone, specimens made of a single type of recycled aggregate often have a higher elastic modulus. When different types of aggregates are mixed, the prepared materials may exhibit unique mechanical properties and stress-strain curve characteristics due to the interaction between them.

It can also be seen from the curve in Fig 6 that regardless of the replacement rate of recycled aggregate, the peak strength of concrete generally increases with the extension of curing age (from 7 days to 28 days). This shows that appropriately extending the curing time can still increase the strength of recycled aggregate concrete to a certain extent. Meanwhile, when using recycled aggregate to replace graded crushed stone, it should not be considered according to the concept of traditional recycled aggregate replacement of graded crushed stone, but should fully consider the source of recycled aggregate and initial strength. For recycled aggregates with high strength and good performance, a high replacement rate should be used to help recycle resources and have a positive effect on the mechanical properties of concrete. In addition, it can be seen from Figs 5 and 6 that the mechanical properties of recycled aggregate concrete are comprehensively affected by the amount of soil cement, the replacement rate of recycled aggregate, and the curing age. In actual engineering applications, to optimize the performance of recycled aggregate concrete, these factors must be considered comprehensively to select appropriate mix proportions and curing strategies. This can not only improve the mechanical properties of the material but also achieve efficient use of resources in the context of sustainable development. Therefore, the relationship between the mechanical properties of recycled aggregate concrete and multiple factors will be further quantitatively explored in the future.

### Compressive strength analysis

**Fig 7:**
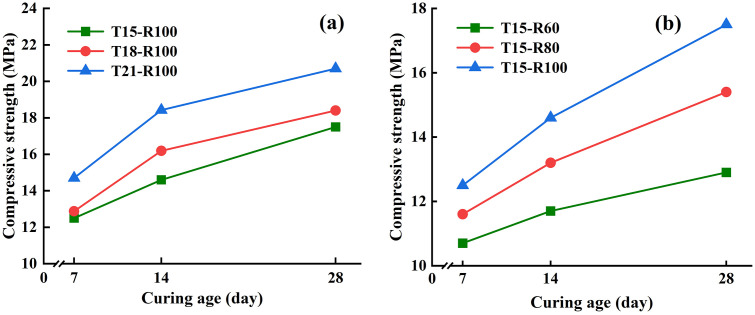
Distribution curve of compressive strength of recycled aggregate concrete with curing age. (a) Different soil cement content; (b) Different recycled aggregate replacement rates.

The cured recycled aggregates with different soil cement content and different recycled aggregate replacement rates showed significant differences in the compressive strength test, as shown in Fig 7. Fig 7(a) shows the average change trend of the compressive strength of recycled aggregate concrete with curing age (7 days, 14 days, and 28 days) under three different soil cement content. Specifically, when the soil rock content is 15%, the 7-day compressive strength is about 12.5 MPa, increases to about 14.6 MPa at 14 days, and reaches about 17.5 MPa at 28 days. The compressive strength growth trend is relatively slow. When the soil cement content reaches 18%, the 7-day compressive strength is about 13.0 MPa, increases to about 16.0 MPa at 14 days, and reaches about 18.4 MPa at 28 days. Compared with the soil cement content of 15%, the compressive strength is higher at all time points. When the amount of soil cement content increases to 21%, the compressive strength is about 14.69 MPa at 7 days, about 18.42 MPa at 14 days, and close to 21 MPa at 28 days, showing the highest strength growth. In general, with the increase of soil cement content, the compressive strength of recycled aggregate concrete increases significantly, indicating that the amount of soil cement content has a positive effect on the compressive properties of recycled aggregate concrete.

In addition, Fig 7(b) shows the effect of different recycled aggregate replacement rates on the compressive strength of concrete under the condition of fixed soil cement content (T15). The compressive strength of concrete with a recycled aggregate replacement rate of 60% is 10.7 MPa at 7 days, 11.7 MPa at 14 days, and 13 MPa at 28 days, and the strength growth is relatively slow. When the recycled aggregate replacement rate is 80%, the strength growth rate of recycled aggregate concrete is significantly higher than that of T15-R60. Concrete with a recycled aggregate replacement rate of 100% shows the highest strength growth. This result differs from most conventional studies, in which higher recycled aggregate contents usually reduce mechanical performance. The reason is that the recycled aggregates used in this study originated from long-serving reinforced-concrete bridge structures, which possessed high original strength, low porosity, and dense internal microstructure. These characteristics allow the bridge-grade recycled aggregate to retain superior mechanical integrity even after crushing, thereby enhancing the overall load-bearing capacity of the recycled aggregate concrete matrix. As a result, the 100% recycled aggregate mixture still exhibits improved compressive strength, further confirming the high quality and structural compactness of the bridge-demolition aggregates.

As can be seen from the above, with the increase of curing age, the compressive strength of all types of recycled aggregate concrete has increased significantly. Whether it is an increase in the amount of soil cement content or an increase in the replacement rate of recycled aggregate, the compressive strength of recycled aggregate concrete can be effectively enhanced. Therefore, in practical applications, optimizing the selection of soil cement content and recycled aggregate replacement rate can effectively improve the mechanical properties of recycled aggregate concrete, thereby meeting the needs of different projects. These results are of great significance for the development of green building materials and for improving resource utilization.

### Multi-factor mechanical response model analysis

**Fig 8:**
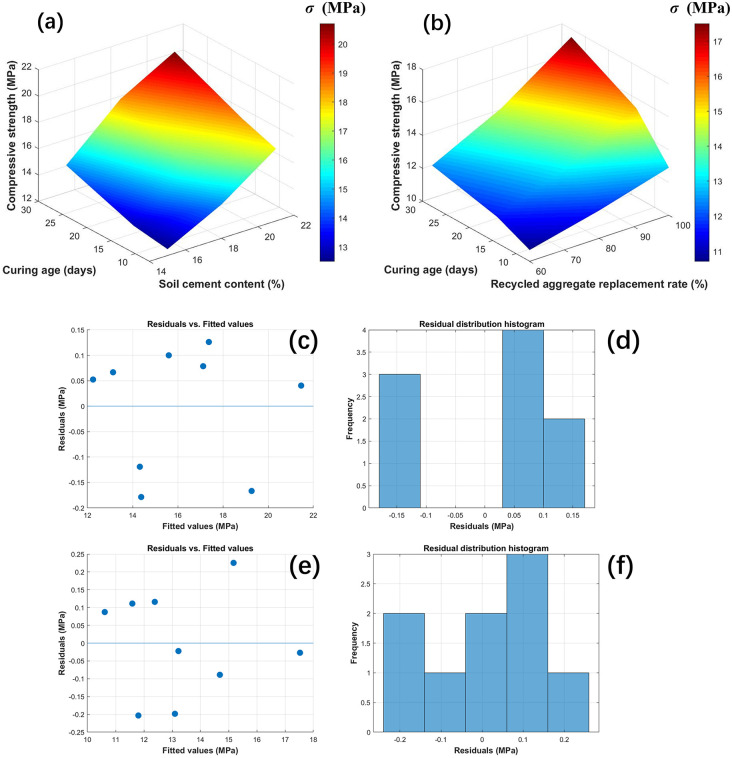
Three-dimensional fitting surface between soil cement content (recycled aggregate replacement rate), curing age, and compressive strength of recycled aggregate concrete. (a) 100% recycled aggregate replacement rate; (b) 15% soil cement content; (c) (e) Figs 8 (a) and (b) show the residual points of the fitted surfaces in the “predicted value - residual” scatter plot; (d) (f) The residual histograms of the fitted surfaces in Figs 8 (a) and (b) are shown.

The mechanical properties of concrete are affected by many factors, including raw materials, proportions, and curing age. In this study, the effects of soil cement content, recycled aggregate replacement rate, and curing age on the compressive strength of recycled aggregate concrete were analyzed (Figs 5-7). Based on the above analysis, this section uses MATLAB programming to perform a three-dimensional fitting surface on the relationship between soil cement content, recycled aggregate replacement rate, curing age, and the compressive strength of recycled aggregate. Fig 8 shows the three-dimensional fitting surface diagram of compressive strength under different conditions. Through these diagrams, the interaction between various factors and their comprehensive influence on compressive strength can be intuitively observed.

As shown in Fig 8(a), the compressive strength of recycled aggregate concrete under the condition of 100% recycled aggregate replacement increases with the increase in soil–cement content and curing age. Specifically, with increasing soil–cement content, the compressive strength shows a significant upward trend. This is mainly because an appropriate amount of soil–cement can improve the internal microstructure of concrete and enhance bonding between particles. In addition, regardless of whether the soil–cement content is low (15%) or high (21%), the compressive strength increases notably as the curing age extends from 7 to 28 days. Prolonged curing age promotes continued hydration reaction and thus contributes to strength development.

Fig 8(b) shows the effect of different replacement rates of recycled aggregate on compressive strength under a fixed soil–cement content of 15%. By analyzing the surface, it can be seen that the compressive strength increases as the replacement rate increases from 60% to 100%. For example, when the replacement rate increases from 60% to 80% at 28 days, the compressive strength increases by 16.80%; when the replacement rate increases from 80% to 100%, the strength increases by 19.86%. This indicates that at higher replacement rates, the mechanical quality and integrity of recycled aggregate contribute more positively to the strength of the concrete. Meanwhile, even at relatively low replacement rates, increasing the curing age effectively improves compressive strength, suggesting that optimized curing conditions can mitigate strength loss associated with lower-quality recycled aggregate.

To more accurately describe and predict the coupled influence of these factors, we developed a multivariate polynomial regression model to fit the experimental data. The complete regression formulas are shown in Eqs. (1) and (2), where all variables and coefficients are clearly defined to ensure full model transparency and reproducibility. Moreover, the MATLAB code used for model fitting has been provided in the revised manuscript, including data preprocessing, fitting commands (fitlm/poly22), and parameter extraction, allowing other researchers to fully reproduce the regression process.


$$\sigma =\alpha \;\cdot \omega^{\text{2}}+\beta \;\cdot \chi^{\text{2}}+\gamma \;\cdot \omega \cdot \chi +\delta \;\cdot \omega +\lambda \;\cdot \chi +\sigma_{\text{0}}\;$$ (1)

$$\sigma =\alpha \;\cdot \eta^{\text{2}}+\beta \;\cdot \chi^{\text{2}}+\gamma \;\cdot \eta \cdot \chi +\delta \;\cdot \eta +\lambda \;\cdot \chi +\sigma_{\text{0}}\;$$ (2)

where, *σ* is the compressive strength, MPa; *ω* is the soil cement content, %; *η* is the replacement rate of recycled aggregate, %; *χ* is the curing age, d; *α*, *β*, *γ*, *δ* and *λ* are the polynomial surface coefficients, and *σ*_0_ is the constant term. The specific parameters are shown in Table 4.

As shown in Fig 8 and Table 4, the models exhibit excellent fitting accuracy, with *R*^2^ values of 0.9984 and 0.9953, respectively. In addition to fitting accuracy, a detailed residual distribution analysis has been added to the revised manuscript. The residual–prediction scatter plots and residual histograms (Fig 8(c)-(f)) show that the residuals are randomly and symmetrically distributed around zero, with no clustering or systematic deviation. The absolute residual values fall within ±5% of the measured compressive strength, indicating that the model has no structural bias and possesses stable predictive performance. This confirms that the polynomial regression model reliably captures the interaction between factors and provides accurate predictions of compressive strength.

Furthermore, we added a subsection discussing model applicability. The regression equations are applicable within the tested parameter ranges: soil–cement content 15–21%, recycled aggregate replacement 60–100%, and curing age 7–28 days. Outside these ranges—such as extremely low replacement rates or extended curing periods—additional calibration or higher-order models may be required. In summary, the enhanced multi-factor regression analysis provides a transparent, reproducible, and statistically validated description of the interactions among soil–cement content, recycled aggregate replacement rate, and curing age. The results offer a theoretical basis for optimizing mix design and improving the performance of recycled aggregate concrete.

## Analysis of microstructure characteristics of recycled aggregate concrete

**Fig 9:**
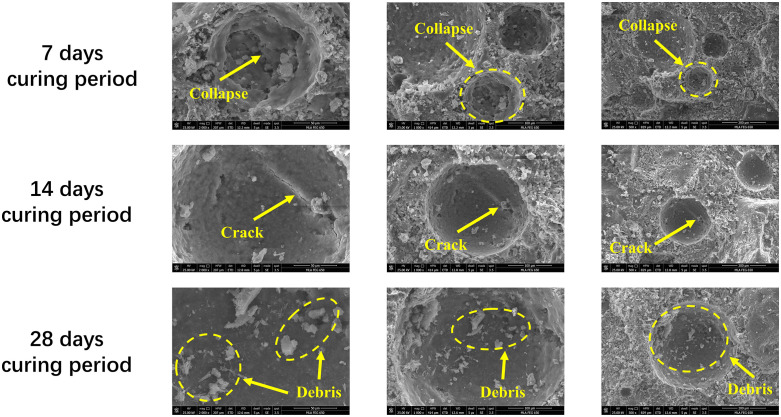
SEM microscopic images of recycled aggregate concrete at different curing ages (T15-R100). Note: Yellow arrows and dashed circles indicate typical features such as collapse zones, cracks and loose debris. Hydration products including dense C–S–H gel, ettringite needles and portlandite crystals can be observed in the interfacial transition zone and paste matrix, illustrating the progressive densification of the microstructure and refinement of the pore system with increasing curing age.

Fig 9 shows the SEM microscopic images of recycled aggregate concrete made with 15% soil cement content and 100% recycled aggregate replacement rate at different curing ages (7 days, 14 days, and 28 days). Among them, the SEM image is the result of sampling from the centre of the concrete after external load and scanning by electron microscope, reflecting the internal microstructural characteristics of recycled aggregate concrete under external force. In addition to pores and cracks, the main hydration products visible in Fig 9 include dense C–S–H gel surrounding the aggregate and filling the matrix, plate-like portlandite crystals and acicular ettringite needles distributed in the interfacial transition zone (ITZ) and paste matrix.

At the curing age of 7 days (Figs 9(a)-(c)), the microstructure of the concrete showed obvious pores and almost complete collapse. The image magnified 2000 times (Fig 9(a)) shows a significant collapse area, indicating that under external force loading, the interface transition zone is still in a weakened state, and the bonding force between the aggregate and the matrix is insufficient, resulting in the local structure being easily unstable under external force. The C–S–H gel in this stage appears relatively loose and non-uniform, and a considerable amount of unhydrated or partially hydrated clinker particles can still be observed. Needle-like ettringite is mainly concentrated in the ITZ and along the pore walls, which, together with the large pores, results in a highly discontinuous microstructure. Similar pores and collapsed areas can also be observed in the 1000x and 500x images (Figs 9(b) and (c)), which indicate that recycled aggregate concrete is prone to significant local damage after external force loading at an early age, and the compressive strength is insufficient to resist the external force. At the 14-day curing age (Figs 9(d)-(f)), the microstructure of recycled aggregate concrete shows more obvious crack propagation. In the image magnified 2000 times (Fig 9(d)), fine cracks can be seen extending outward from the interface transition zone. These cracks further expand under the action of external force, affecting the integrity of the overall structure. Compared with 7 days, the amount of C–S–H gel increases and begins to form a more continuous network that partially fills the original pores around the recycled aggregate. Ettringite is still present in the ITZ as needle or rod-like crystals, but its distribution becomes more dispersed, and the volume of large portlandite plates decreases, indicating that hydration continues to consume portlandite and generate secondary C–S–H. The 1000x and 500x magnified images (Figs 9(e) and (f)) show larger-scale cracks that run through the interface between the new and old aggregates, indicating that during the 14-day curing process, although the bonding has improved, the microstructure still has weak links under external force loading, showing a certain tendency to crack propagation. At the 28-day curing age (Figs 9(g)-(i)), the microstructure of the recycled aggregate concrete was significantly improved. The 2000-fold magnification (Fig 9(g)) shows that the structure in the interface transition zone is more compact, and the debris and cracks are significantly reduced, which means that the internal structural stability of the concrete is improved after a longer curing age and external force loading. The C–S–H gel becomes dense and uniformly distributed, closely wrapping the recycled aggregate and effectively bridging the ITZ, while the amount of free ettringite needles and large portlandite crystals is further reduced. Many of the pores observed at 7 and 14 days are now partially or completely filled by hydration products, leading to a refined pore structure and fewer continuous crack paths. The 1000-fold and 500-fold magnification images (Figs 9(h) and (i)) show that the 28-day recycled aggregate concrete has almost no cracks after being loaded, and the microcracks tend to close, which corresponds to the higher compressive strength (Section *Compressive strength analysis*).

In summary, the microstructure of recycled aggregate concrete shows obvious changes under the combined action of external force loading and different curing ages. At the early age (7 days), the structure becomes loose under load, the pore collapse phenomenon is serious, and the compressive capacity is insufficient; at the middle age (14 days), the cracks extend significantly, and the external force loading aggravates the fragility of the microstructure; while at the late age (28 days), the microstructure is dense, and cracks hardly appear even under the action of external force, indicating that its compressive capacity is higher. This evolution is closely related to the development of hydration products: from 7 to 28 days, the continuous formation and densification of C–S–H gel, together with the refinement and redistribution of ettringite and the reduction of large portlandite crystals and capillary pores, leads to a more homogeneous and compact ITZ and paste matrix. These changes provide a microscopic explanation for the observed increase in compressive strength and improved crack resistance with curing age. These microstructural characteristics directly affect the macroscopic mechanical properties of recycled aggregate concrete, reveal the mechanism of the influence of external force on the evolution of its microscopic damage, and provide a scientific basis for optimizing mix design and improving the durability of recycled aggregate concrete.

## Analysis of concrete structure evolution and microscopic mechanism of mechanical properties

**Fig 10:**
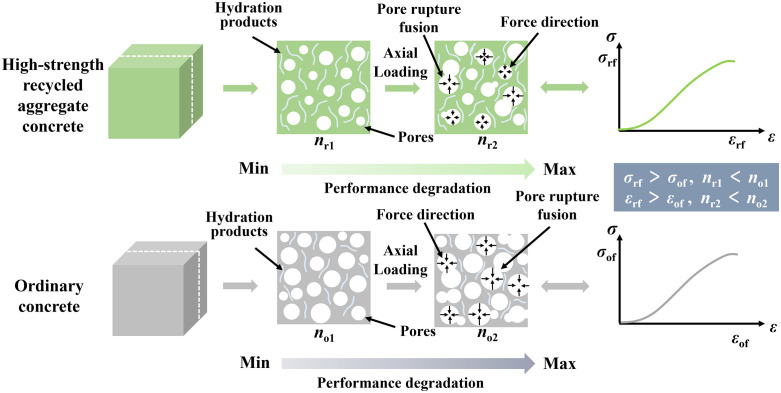
Schematic diagram of strength formation and pore structure evolution of recycled aggregate concrete and ordinary concrete under loading.Note: OC prepared with 100% natural aggregates and conventional mix proportions, used as the reference material in this study.

Recycled aggregate concrete (RAC) exhibits significant differences from ordinary concrete (OC) in terms of microstructural characteristics and mechanical performance. In this study, OC refers to a reference concrete prepared with 100% natural coarse and fine aggregates using conventional mix proportions, and is used as the benchmark material for comparison with RAC; it does not represent a low-quality recycled aggregate concrete or any other hypothetical material. These differences mainly stem from the unique properties of recycled aggregate and its role within the concrete matrix. Typically, under external loading, the deterioration of concrete strength involves microstructural changes, including damage to the pore structure, degradation of the interfacial transition zone (ITZ), and the propagation of internal microcracks [25]. These microscopic mechanisms often lead to substantial reductions in the macroscopic mechanical properties of concrete. However, the findings of this study indicate that under specific conditions involving high-strength recycled aggregates, the compressive strength of concrete is not adversely affected. On the contrary, with increasing replacement ratios of recycled aggregate, the strength improves under identical conditions (Fig 7). This phenomenon deviates from conventional understanding [16,23], making it necessary to conduct an in-depth analysis from the perspective of microstructural evolution and material properties to reveal the underlying mechanism of strength enhancement. Based on the results of this study and related literature [26–28], Fig 10 presents a conceptual schematic comparing RAC with OC in terms of strength development, changes in pore structure during loading, and performance degradation. In the figure, *n* represents porosity, *σ* represents stress, and *ε* represents strain.

As shown in Fig 10, RAC forms dense hydration products through the hydration reaction, which fills the voids between aggregates. Although recycled aggregates may contain certain defects (such as microcracks and adhered mortar), the relatively lower porosity within RAC allows hydration products to effectively fill these voids, thereby reducing overall porosity and enhancing the concrete’s strength. During axial loading, the pores in RAC tend to rupture and merge, forming a more compact structure. This is reflected in the stress-strain curve as a higher peak stress (*σ*_rf_) and greater peak strain (*ε*_rf_). In contrast, OC contains relatively fewer hydration products and has a higher initial porosity. During axial loading, these pores are more susceptible to rupture and propagation, resulting in rapid degradation of mechanical performance. The pore structure in OC is generally looser, and after pore rupture, it is difficult to form a dense structure, leading to lower peak stress (*σ*_of_) and peak strain (*ε*_of_) in the stress-strain response, thereby exhibiting inferior mechanical properties. Moreover, the use of high-strength recycled aggregates helps to resist strength degradation to some extent. This is mainly attributed to the incorporation of high-density and high-bond-strength aggregates, which enhance the structural integrity of the concrete at the microscale. Under sustained external loading, traditional concrete typically under-goes a process of crack evolution from microcracks to macrocracks. In contrast, RAC, due to its denser internal structure and stronger bonding, can effectively inhibit microcrack propagation and delay crack coalescence (Fig 10). This retarding effect enables RAC to maintain a high strength level even under high-stress conditions, thereby avoiding common issues related to strength deterioration.

In summary, RAC can effectively enhance the overall mechanical performance of concrete by optimizing the pore structure and increasing the density of hydration products. This improvement is particularly evident under high-strength conditions. The optimized pore structure enables RAC to better resist performance degradation caused by pore rupture and fusion during axial loading, thereby maintaining a higher strength level.

## Discussion

The present study shows that the behavior of soil-cement–solidified recycled aggregate concrete (RAC) prepared with bridge-demolition aggregates is controlled by the coupled effects of recycled aggregate quality, soil-cement dosage, and curing age. In contrast to the conventional understanding that increasing recycled aggregate replacement generally leads to strength reduction due to higher porosity and weak adhered mortar layers, many of the mixtures in this work exhibited increasing compressive strength and enhanced crack resistance at higher replacement ratios. This deviation reflects the role of high-strength parent concrete, which yields recycled aggregates with low intrinsic porosity and robust mechanical integrity, and highlights that the performance of RAC cannot be assessed solely based on replacement level, but must be interpreted in conjunction with the characteristics of the source concrete and the binder system used [29–31].

From the perspective of physical and mechanical properties, the stress–strain curves and crack-morphology evolution (Section *Physical and mechanical properties of soil cement solidified recycled aggregate concrete*) reveal a progressive transition from single wide cracks at 7 days to dense networks of fine microcracks at 28 days, accompanied by substantial gains in peak stress and peak strain. This behavior is consistent with the strengthening of the interfacial transition zone (ITZ) and matrix cohesion as the soil-cement dosage increases from 15% to 21%, leading to improved load transfer and a more ductile failure response. The observation that 100% recycled aggregate replacement yields the highest compressive strength contradicts results obtained for RAC produced from low-quality construction and demolition waste, where higher replacement typically degrades stiffness and strength [32,33]. However, it aligns with recent findings that RAC made from high-strength parent concrete can attain equal or superior mechanical properties to natural-aggregate concrete when mix design and curing are properly controlled [29,30]. The multivariate regression model developed in this study further confirms that soil-cement content, replacement ratio and curing age interact non-linearly, and that strength enhancement is maximized when high-quality recycled aggregates are combined with sufficient soil-cement to densify the matrix, providing a quantitative basis for mix-proportion optimization.

The SEM observations (Section *Analysis of microstructure characteristics of recycled aggregate concrete*) provide a microstructural explanation for the macroscopic strength development and crack evolution. At 7 days, the loose C–S–H gel, abundant unhydrated particles and large connected pores near the ITZ result in local collapse and wide cracks under loading; by 14 days, a denser C–S–H network and partially filled pores reduce crack width but still allow crack propagation along weak ITZ regions. At 28 days, the ITZ becomes significantly more compact, with dense C–S–H, reduced portlandite plates and more finely dispersed ettringite, leading to refined pore structures and limited crack continuity. This pattern is consistent with microstructural studies on RAC, which report that progressive hydration and secondary reactions consume portlandite, generate additional C–S–H and refine pore networks, thereby enhancing microhardness and elastic modulus at the ITZ [34, 35]. Similar densification and improvement of bonding have also been observed in soils and sludge stabilized with industrial-waste–based or calcium-based binders, where intertwined C–S–H and ettringite frameworks markedly increase compressive strength and resistance to microcracking [36]. These parallels support the interpretation that soil-cement in this study plays a comparable microstructural role, promoting matrix densification and ITZ strengthening around bridge-derived recycled aggregates.

Linking these microstructural changes to the conceptual comparison between RAC and ordinary concrete (Section *Analysis of concrete structure evolution and microscopic mechanism of mechanical properties*) clarifies the mechanism by which high-quality recycled aggregates and soil-cement jointly enhance performance. Ordinary concrete with natural aggregates typically exhibits a looser pore structure and a thinner, more porous ITZ; under axial loading, pores tend to open and coalesce, and microcracks rapidly evolve into macrocracks, leading to earlier stiffness degradation and lower peak stress. In contrast, the bridge-derived RAC in this study benefits from aggregates with dense internal microstructure and rough, well-bonded surfaces, which promote the accumulation of hydration products at the ITZ and reduce the prevalence of weak interfacial zones. Recent reviews on RAC emphasize that when recycled aggregates are sourced from high-grade structural concrete and combined with optimized binder systems, the resulting RAC can show mechanical and durability performance comparable to or exceeding conventional concrete, especially in terms of compressive strength, modulus and crack resistance [29,30]. The present findings therefore support the view that RAC performance is not intrinsically inferior, but highly dependent on parent concrete quality, binder composition and curing regime, and they provide experimental and microstructural evidence that soil-cement–solidified RAC using bridge demolition waste can be a structurally reliable and environmentally beneficial material for infrastructure applications.

## Conclusions

This study investigated the mechanical behaviour and microstructural evolution of recycled aggregate concrete (RAC) prepared using high-strength recycled aggregates derived from dismantled bridge structures. Through CS testing, SEM characterization, and multivariate regression modelling, several key findings and insights were obtained.

(1) Summary of major findings

The compressive strength of RAC increased with higher recycled aggregate replacement ratios and appropriate soil–cement contents. The mixture containing 100% recycled aggregate and 21% soil–cement content exhibited the highest strength. Strength development remained highly time-dependent, with significant gains observed between 7 and 28 days. SEM analysis revealed progressive microstructural densification, transitioning from early-age pore-crack networks to a compact matrix at later ages. The proposed multivariate polynomial regression model successfully captured the combined effects of soil–cement content, replacement ratio, and curing age, showing strong predictive accuracy and good agreement with experimental results. These findings highlight that high-quality recycled aggregates sourced from dismantled bridges possess the mechanical integrity necessary for structural concrete applications and can effectively replace natural aggregates.

(2) Critical analysis

Although the results confirm the feasibility of producing high-performance RAC using bridge-grade recycled aggregates, the mechanical improvement is strongly dependent on the intrinsic quality of the parent concrete. The performance enhancements observed in this study are attributable to the superior crushing resistance and angularity of the bridge-derived aggregates, which may not be representative of recycled materials obtained from ordinary building demolition or mixed-source construction waste. Additionally, the positive interaction between soil–cement content and curing age indicates that the binder system plays an important role in compensating for the weaker interfacial transition zone (ITZ) commonly associated with recycled aggregates. This highlights the need for mix-design optimization tailored to the specific properties of the recycled materials rather than relying on universal proportions.

(3) Limitations

Despite the robustness of the experimental programme, several limitations should be acknowledged: Firstly, all recycled aggregates were sourced from dismantled bridge structures with consistently high strength. The findings may not apply directly to recycled materials with lower or more variable quality. Secondly, the study investigated soil–cement contents between 15–21%, replacement ratios between 60–100%, and curing ages of 7–28 days. Behaviour outside these ranges remains uncertain. Thirdly, the polynomial regression model is data-driven and may not fully capture non-linear behaviours under extreme mix designs or environmental conditions. These limitations indicate that although the conclusions are reliable within the tested domain, broader generalization requires further validation.

(4) Recommendations for future research

To strengthen the engineering applicability of RAC prepared from demolition waste, future studies should focus on: Extending mix-design variables to include wider ranges of replacement ratios, soil–cement contents, and additional binder types (e.g., fly ash, slag, calcined clays). Investigating long-term durability under cycles of drying–wetting, freeze–thaw, sulphate attack, and carbonation, especially for full-scale structural applications. Developing performance-based classification systems for recycled aggregates from mixed demolition sources to support standardized high-value utilization. Integrating constitutive modelling with micromechanical simulation or DEM to better understand fracture mechanisms and ITZ evolution. Validating the regression model with larger datasets, field-scale RAC structures, and machine-learning-based predictive tools for enhanced generalization.

## Supporting information

S1 File Code-Fig3&4. (TXT)

S2 File Code-Fig8a. (TXT)

S3 File Code-Fig8b. (TXT)
